# Recent advances in the management of large and complex colonic polyps

**DOI:** 10.12688/f1000research.12930.1

**Published:** 2018-03-12

**Authors:** Gaius Longcroft-Wheaton, Megha Bhandari, Asma Alkandari, Pradeep Bhandari

**Affiliations:** 1 Department of Gastroenterology, Portsmouth Hospitals NHS Trust, Queen Alexandra Hospital, Southwick Hill Road, Cosham, Portsmouth PO6 3LY, UK; 2 Department of Pharmacy and Biomedical Sciences, University of Portsmouth, Winston Churchill Avenue, Portsmouth P01 2UP, UK

**Keywords:** colonic polyps, complex polyps, endoscopic management, Colorectal cancer

## Abstract

The endoscopic management of large colonic polyps is a rapidly changing field. Rapid evolution in endoscopic techniques and skills has resulted in diminishing the role of surgery in the management of larger and complex polyps. This is resulting in organ preservation for many who otherwise would have undergone surgery. However, it also poses new challenges. This article reviews these new advances and the developments which are overcoming these difficulties.

## Introduction

Colorectal cancer is the fourth leading cause of cancer death in the UK, responsible for 15,903 deaths in 2014. Despite this, 54% of cases are preventable ^1^. This is because cancers develop predominately through the adenoma carcinoma sequence and for a long time are benign adenomatous polyps ^2^. Some cancers in the right colon develop along different pathways, but again these exist for a long time as benign serrated polyps (sessile serrated polyps) before undergoing malignant transformation. Removal of these reduces cancer risk, and data from bowel cancer screening programmes have shown that the risk of cancer death can be reduced through screening and adequate resection of polyps ^3, 4^. Whilst over 90% of all polyps are small (less than 10 mm in size), it is not uncommon to find large benign or early neoplastic lesions. These can be 40 mm or more in size. The morphology of these lesions can be accurately described by using the Paris classification system, which classifies lesions as pedunculated (1p), sessile (1s), or flat (iia, iib, iic) or, for larger lesions, as laterally spreading tumours granular (LST-G) or non-granular (LST-NG). This can give some prediction of the risk of invasive disease, and non-granular or flat depressed (iic) lesions carry a high risk of invasive cancer ^5^. Likewise, assessments of surface and vascular patterns can be used to predict lesion histology and, in particular, assess for areas of early invasion ^6^. Furthermore, difficult access, the presence of scarring, and location close to the dentate line, appendical orifice, ileo-caecal valve, or diverticula can make resection very challenging, and resection of these difficult polyps requires techniques beyond simple polypectomy and also poses new risks of complications. These risks can be predicted by using classification systems such as the size/morphology/site/access (SMSA) scoring system ^7^. In this review, we will discuss techniques for the resection of these complex polyps and measures that can be taken to minimise risks.

## Endoscopic mucosal resection

Endoscopic mucosal resection (EMR) involves the injection of fluid into the submucosal space to lift the lesion away from the muscle and resection in single or multiple (>2 cm in size) pieces by using an electrocautery snare. This technique revolutionised the management of large and flat polyps. Endoscopists have started removing larger and larger lesions with this technique. Studies have demonstrated that this technique is more cost effective than surgery, and a large Australian study suggested that a mean cost saving of $7,602 USD per patient could be achieved ^8^.

Large trials have shown the clinical benefits of this, and the Australian Colonic EMR (ACE) study of 1,134 patients from Australia ^9^ and a study of 220 patients from the UK ^7^ both demonstrated that excellent results can be achieved. A recent meta-analysis reviewed all of the literature on polyps measuring more than 2 cm in size and resected by an endoscopic approach. This large analysis of EMR examined 50 studies, including 6,442 patients and 6,779 large polyps (over 20 mm in size) ^10^, and demonstrated an initial success rate of 92%, and 503 patients underwent surgery for a non-curative endoscopic resection. Perforation and bleeding rates of 1.5% and 6.5%, respectively, were observed. Recurrence at follow-up was detected in 13.8% of patients. Overall, endoscopic treatment was successful in 90.3% of cases. Polyp cancer was found in 8% of cases. Recently, the European Society of Gastrointestinal Endoscopy (ESGE) recommended that the majority of colonic and rectal lesions can be effectively removed in a curative way by EMR ^11^.

These data illustrate the strength of EMR, which prevented surgery in 90% of patients but does have limitations in terms of recurrence and management of superficial polyp cancers. The keys to success are lesion recognition skills, training, and experience. The ESGE recommends that lesions of more than 20 mm in size which are sessile, laterally spreading, or complex are removed by an appropriately trained, experienced endoscopist in a specialist centre.

## Endoscopic submucosal dissection

**Figure 1.**
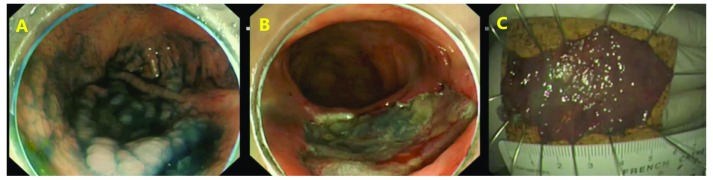
Endoscopic submucosal dissection. The lesion is initially assessed and submucosal lifting performed ( **A**) before a complete resection is performed ( **B**). The lesion is resected en-bloc before being pinned out and sent for histopathological examination ( **C**).

When the polyps are larger than 20 mm in size and especially flat, the risk of cancer in these polyps increases and resection by EMR technique results in multi-piece resection, which is considered to be associated with a high risk of recurrence ^12^. Endoscopic submucosal dissection (ESD) is an oncologically superior technique designed to resect lesions in an en-bloc fashion. It involves submucosal injection followed by the use of an ESD knife to perform mucosal incision, followed by trimming of the submucosal edges to allow access to the submucosal plane. This is followed by full submucosal dissection resulting in an en-bloc resection ( Figure 1). This technique is well established in Japan, where it was initially developed for the resection of early gastric cancers. The advantages of ESD are related to en-bloc resection; this allows histological confirmation of complete resection (R0), low recurrence, and a complete cure for low-risk superficial neoplasia. Studies have demonstrated that colorectal ESD has very low recurrence rates (under 2%) ^13^. However, complications, particularly perforations, which occur in around 5% of cases, are more frequent. The learning curve for ESD is long and steep outside Japan because of a lack of trainers and training facilities.

ESGE guidelines recommend that in cases where there is a high suspicion of submucosal invasion and an en-bloc resection cannot be achieved with an EMR technique, ESD is considered ^11^. This is particularly important if there is a suspicion of a iic depressed area or type V surface pit patterns. Recent systematic review and meta-analysis reviewed 97 studies with 18,764 colorectal lesions resected by standard ESD technique and reported a pooled R0 resection rate of 82.9% (95% confidence interval [CI] 80.4–85.1%) and an en-bloc resection rate of 91% (95% CI 89.2–92.5%) ^14^. They reported a very low complication rate with delayed bleeding rate at 2.7% (CI 2.2–3.2%), perforation at 5.2% (CI 4.4–6.1%), and recurrence at 12 months of 2% (CI 1.3–3.0%). This study further analysed the results on the basis of Asian and non-Asian (Western) studies and found some interesting differences. Of note, there was a significantly poor outcome and higher complication rates in Western settings. We believe that this is due to poor training and lack of a formal accreditation process in ESD competence. Until we develop a good training structure or make the technique safer, ESD in the West should be restricted to experts at high-volume centres.

## Hybrid endoscopic submucosal dissection or knife-assisted snare resection

Classic ESD in the West is difficult to learn, takes a long time to perform, and has higher complication rates. A potential compromise solution is a hybrid technique involving components of EMR that are well established in the West and ESD. It is also called knife-assisted snare resection (KAR), in which an ESD knife is used to make a circumferential mucosal incision around the lesion followed by snare resection, in either a single piece (small lesions or after a significant amount of submucosal dissection) or multiple pieces (large lesions or little or no submucosal dissection). This technique has many advantages. As the initial mucosal incision is in the normal mucosa around the lesion, it clearly demarks the resection zone. This reduces the risk of recurrence. KAR involves much less resection as compared with traditional ESD, so this makes it quicker and technically easy for Western endoscopists. KAR has also been used in the resection of very challenging scarred polyps (recurrence following previous EMR). A recent series of 42 patients reported the use of KAR for the resection of very complex scarred lesions with a mean size of 40 mm. The investigators reported that more than one attempt was required for complete clearance of lesions in 35% of patients but that ultimately surgery was avoided in 90% of patients ^15^.

This looks very promising, but a recent meta-analysis reported that R0 and en-bloc resection rates with the hybrid technique were significantly lower than those achieved with the standard technique (60.6% and 68.4%, respectively) and had similar adverse event rates ^14^. This questions the role of this hybrid technique. We feel that it is unfair to compare the outcome of this technique with the outcome of ESD and that it would be better to compare this technique with EMR.

## Making resection safer: management of complications of endoscopic resection

Complications represent a barrier to the endoscopic resection of large benign polyps. These can be broadly divided into immediate events, which occur during the procedure, and delayed events, which can occur up to 4 weeks after the patient has gone home. This includes delayed perforation and delayed bleeding.

Intra-procedural bleeding is typically controlled with thermal energy from the diathermy unit. There are numerous techniques for achieving this, including the tip of the snare or dedicated coagulation forceps. The optimum settings for the delivery of electrical energy have been the subject of research. It is clear that for resection a blended cutting current should be used and that ****pure coagulation should be avoided, as it causes a deep thermal injury and increases the risk of perforation ^14^. Likewise, a pure cutting current, whilst carrying a low risk of perforation, carries a high risk of immediate bleeding ^15^. Once a vessel has started bleeding, it is necessary to employ a technique to achieve haemostasis. A study of 196 patients suggested that soft coagulation using the snare tip could achieve haemostasis in 91% of cases and that coagulating forceps or clips are required in the remaining cases ^16^.

It has been questioned whether prophylactic clip closure of the EMR/ESD deficit could reduce the risk of delayed complications. A study of 524 polyps 2 cm or larger from the United States suggested that the risk of delayed post-polypectomy bleeding could be reduced from 9.7% to 1.8% through full clipping of the deficit ^17^. It should be noted that this was an uncontrolled retrospective study; as a result, the ESGE does not currently recommend routine endoscopic clip closure to prevent delayed bleeding ^11^. However, the same group recently reported a randomised controlled trial (n = 354) and compared post-EMR clip closure with no closure after the resection of medium-sized lesions (1–4 cm). They reported a significant difference in delayed bleeding rates of 6.9% without clip closure as compared with 1.1% after clip closure. This is a compelling piece of evidence, but there has been no cost-benefit analysis ^16, 18^. Given the relatively high cost of clips and the large number required to completely close a broad EMR base, an economic modelling study from Australia has suggested that the expected cost of prophylactic clipping would be €1,106 per lesion compared with a cost of €157 per lesion without clipping and that the funds needed in order to prevent one case of clinically significant bleeding would be €14,826, which the authors concluded was not cost effective ^19^. Furthermore, whilst clipping is safe in experienced hands, there is risk when applying to a mucosal deficit of causing a perforation. The technique required is quite different to their use in obtaining haemostatic control and it is therefore important that, if this approach is to be considered, supervised training in the technique must be obtained. We feel that this will be a highly desirable approach in high-risk patients who have multiple co-morbidities and who are likely to require anti-platelet or anti-coagulant drugs immediately after the procedure.

## Making resection more effective: reducing recurrence risk after endoscopic mucosal resection

There have been a number of proposed methods for reducing recurrence after EMR. It has been questioned whether soft tip coagulation of the edges of the resection margin is of value. This is being investigated in the SCAR (Soft Coagulation for the Prevention of Adenoma Recurrence) study (ClinicalTrials.gov identifier NCT01789749) in Australia ^20^. The preliminary results from this randomised controlled trial, published in abstract form only, have suggested that recurrence rates fall from 20.6% to 5.8%. These results should be interpreted with caution, as they have yet to be published, and the recurrence rates in the control limb were much higher than results previously published by the same group where a recurrence rate of 16% was observed ^9^. This could suggest issues with training of the multiple endoscopists in this larger study or lesion selection.

It has been questioned whether performing an extended EMR (X-EMR) could reduce recurrence rates. A study of 471 lesions resected by standard EMR and 448 lesions resected by X-EMR did not show any reduction in residual or recurrent adenoma at follow-up (11.7% versus 10.1%, hazard ratio 0.8, CI 0.5–1.3, *p* = 0.499). However, complications were increased, and there was increased intra-procedural bleeding (odds ratio 3.1, CI 2.0–5.0, *p* <0.001) ^21^. Therefore, it is possible that recurrence in EMR is not coming from the lesion margin but instead from microscopic residual adenoma in endoscopically imperceptible tissue bridges between piecemeal resections within the ulcer base, where the resections overlap, which cannot be identified by visual inspection of the final lesion base. What has become clear, however, is that incomplete resection is associated with an increased risk of lesion recurrence and that this is less likely to occur if the resection is performed by a specialist endoscopist. A retrospective study of 257 patients with 269 polyps demonstrated the protective effect of a specialist endoscopist, and the odds ratio for incomplete resection was 0.13 (CI 0.04–0.41) ^22^. Whilst there will be continuing debate as to what defines an ‘expert’, this is not an unexpected finding, and it is clear that whilst EMR is not as technically demanding as ESD, it should be performed by endoscopists who have been trained in the technique and who perform such resections on a regular basis with audited outcome data.

It is possible to predict the risk of lesion recurrence, and larger polyps carry an increased risk of incomplete resection or lesion recurrence ^7, 22^. Likewise, flat laterally spreading lesions or a poor lift also increases the complexity of resection and increases risk ^7, 22^. The Sydney EMR recurrence tool (SERT) can be used to predict the risk of adenoma recurrence. This identified lesion size, intra-procedural bleeding, and high-grade dysplasia as independent risk factors ^23^. Owing to its nature, it can be applied only post-resection but could be of value in stratifying the most appropriate first surveillance interval. A classification system based on SMSA can be used to risk-stratify lesions (by the degree of complexity) prior to endoscopy and could be of benefit in the assignment of lesions to the most appropriate endoscopist ^7^.

Lesion assessment prior to resection is critical for reducing not only the risk of complications or residual adenoma but also the risk of finding covert cancer within the resection specimen. It is established that in lesions over 2 cm in size this risk increases. This is greatest for non-granular lesions (LST-NG) and those with a depressed component, a combined Paris classification of flat IIa morphology with a bulky Is component, or a high-risk surface pattern. It has also been suggested that a rectosigmoid location carries greater risk ^24^. In lesions at higher risk of covert malignancy, an ESD en-bloc resection is preferable to enable accurate pathological staging and determine whether a cure has been achieved through the endoscopic resection or whether further surgery is needed.

## Futuristic look

One of the difficulties of ESD has been in achieving traction on the lesion. The most commonly taken approach is a transparent hood, or cap, on the end of the endoscope, which enables the lesion to be physically pushed out of the field of dissection. However, research has been conducted in approaches to allow access of tissue retractors to facilitate faster and safer submucosal dissection. A novel system of an expandable working chamber with two independent instrument guides has been used in an *in vivo* model to achieve successful ESD with a faster procedure time and more effective dynamic tissue retraction and instrument triangulation ^25^. It has been suggested that this approach may reduce the learning curve for ESD. In a similar manner, a three-dimensional printed overtube system with two manipulator arms at the tip has been used in an animal study with similar results ^26^. A modification of the transanal minimally invasive surgery (TAMIS) system has been developed for use in ESD ^27^; the modification provides three channels and a sealed system, allowing triangulation, retraction, and control of inflation. A more sophisticated development is the master and slave transluminal endoscopic robot (MASTER) system, which provides a pair of robotic ‘arms’ at the end of the endoscope with an L-shaped hook and grasper ^28^. It should be noted that all of these technologies are in the development phase and are not yet ready for mainstream use but do provide insight into how things may move in the future. A simpler approach to achieving traction is to attach a clip and string to a lesion and apply gentle reaction to open up the submucosal space ^29^. Though crude compared with the previous techniques described, this is being used in current ESD practice in some centres and does not require a significant investment in novel equipment.

## Moving beyond mucosal resection

**Figure 2.**
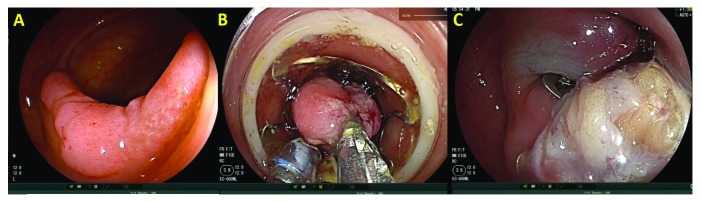
Full-thickness resection. The lesion ( **A**) is pulled into the device ( **B**) before the clip is deployed and resection performed ( **C**).

Traditionally, the whole aim in endoscopic resection has been to avoid a perforation. However, this view is now being challenged with the development of full-thickness resection devices. A specialised device consisting of a transparent cap with a preloaded snare and modified over the scope clip is fitted to a standard colonoscope. Grasping forceps are used to pull the lesion into the cap, after which the clip is deployed. The snare is then closed and electrocautery applied to make a full-thickness resection above the clip ( Figure 2). Early studies have shown success in lesions up to 20 mm in size ^30– 32^. Recently, an endoscopic suturing system (Apollo OverStitch) has become available, which enables full-thickness sutures to be applied. This has been used for the closure of anastomotic dehiscence after colorectal cancer resections ^33^ and, in a case series of 12 patients, was shown to be successful for the closure of conventional ESD defects ^34^. To date, there have been no studies examining its use for full-thickness ESD, but there is no conceptual reason why it should not be effective for such resections, and the possibilities this could open up are intriguing. For scarred lesions where a tissue plane cannot be identified, this may be an attractive option. Likewise, for early cancers, this could provide an alternative to transanal endoscopic microsurgery.

Whilst we have discussed the possibilities of ESD, KAR, and full-thickness resection, all of these techniques are challenging to learn. A device has been developed for shaving such lesions off in multiple pieces; known as EndoRotor, this catheter-based device, passed through the biopsy channel of a standard colonoscope, uses suction to pull in mucosa and a rotating cold blade to cut the tissue before sucking it in and transporting samples to a collection trap. Animal studies have suggested that rapid resection of flat or slightly elevated mucosa is possible ^35^. It is anticipated that the learning curve for achieving confidence will be shorter than that for ESD or KAR, although how it compares with EMR is less certain. Whether it could be used for scarred lesions is unclear and further studies are needed. Because it does not achieve an en-bloc resection, its efficacy, safety, and learning curve should be compared with EMR rather than ESD or full-thickness resection. It is our contention that its future is likely to be dependent on whether it can manage scarred lesions unsuitable for conventional EMR, as the latter is already well established for the resection of unscarred benign pathology in multiple pieces and it is difficult to see how this device could be more effective for such lesions.

## Conclusions

**Figure 3.**
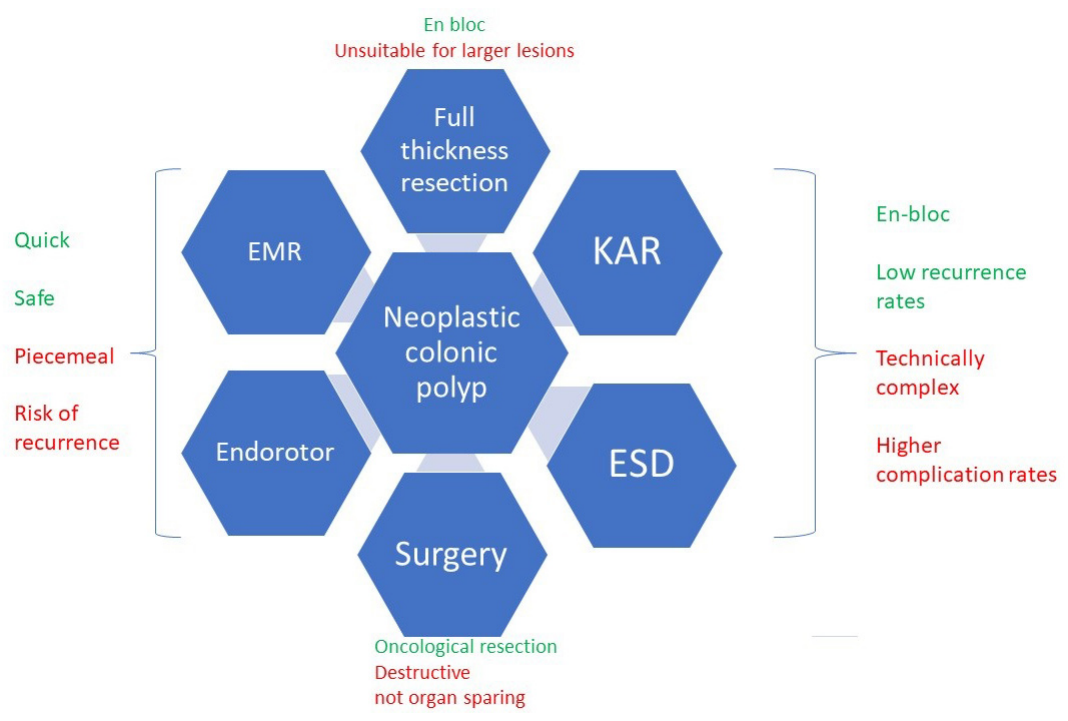
Current options for the resection of colonic neoplasia. EMR, endoscopic mucosal resection; ESD, endoscopic submucosal dissection; KAR, knife-assisted resection.

The last decade has seen a revolution in the management of colonic neoplasia, and there has been a paradigm shift from surgery towards endoscopic resection; the multitude of options are shown in Figure 3. It is our contention that there will be additional significant changes in the way we manage these lesions. There will be a progressive shift away from EMR towards ESD, and hybrid techniques will form a bridge as endoscopists make the shift towards adopting this approach, which carries the advantages of lower recurrence rates and en-bloc resection. EMR will not in any way disappear (as it is a rapid and safe technique) but will be used more for uncomplicated, smaller lesions (under 40 mm in size) where outcomes are excellent. Therefore, case selection will be critical and endoscopists will need to become confident in deciding the best approach for a given lesion. The boundaries of what is considered endoscopically resectable will expand, and there will be a growth in the resection of scarred lesions currently being sent for surgery. Furthermore, it is probable that some early cancers will be resected endoscopically. Many of the problems currently faced by the endoscopist will improve, including haemostasis control, tissue retraction, and tools for managing complications such as perforation. Robotic ESD is some way off but should not be dismissed as fantasy and indeed may be the shape of the future, and organ preservation is more realistic than ever before.

## Abbreviations

CI, confidence interval; EMR, endoscopic mucosal resection; ESD, endoscopic submucosal dissection; ESGE, European Society of Gastrointestinal Endoscopy; KAR, knife-assisted resection; LST-NG, laterally spreading tumour non-granular; SMSA, size/morphology/site/access; X-EMR, extended endoscopic mucosal resection.
